# Heterogeneity of 3’-Untraslated Region of Genome RNA of the Tick-Borne Encephalitis Virus (TBEV) Strains Isolated from Ticks in the Western Siberia, Russia

**Published:** 2007-09

**Authors:** Olga V. Morozova, Valentina N. Bakhvalova, Igor V. Morozov

**Affiliations:** 1 *Institute of Chemical Biology and Fundamental Medicine of Siberian Branch of the Russian Academy of Sciences, Novosibirsk, Russia;*; 2 *Institute of Systematics and Ecology of Animals of Siberian Branch of the Russian Academy of Sciences, Novosibirsk, Russia*

**Keywords:** deletions, hemagglutination, neurovirulence, reverse transcription–polymerase chain reaction, tick-borne encephalitis virus strains, 3’untraslated region sequences

## Abstract

The tick-borne encephalitis virus (TBEV) strains have been isolated from unfed adult ticks *Ixodes persulcatus* Schulze in Novosibirsk region (South-Western Siberia, Russia) beginning from 1980 till 2006. The TBEV 3’-untraslated region (3’UTR) variable fragment was amplified with primers corresponding to conserved flanking areas. The RT-PCR product lengths varied in range from 100 to 400 bp. Comparative analysis of 3’UTR nucleotide sequences revealed a few groups of the TBEV strains within Siberian genetic subtype with significant intra-group homology and essential differences between groups. Correlation between lengths of the 3’UTR fragments and hemagglutination (HA) titers for subsequent passages of the TBEV strains was not found. However, for the viral strains with shorter 3’UTR (less than 200 nucleotides) incubation period for suckling mice was longer than 5 days. It might be resulted from decreased RNA synthesis or reduced neuroinvasiveness.

## INTRODUCTION

Flaviviruses cause a spectrum of serious diseases worldwide. The flaviviral genomes are 5’-capped positive-sense RNA about 11,000 nucleotides long with a nonpolyadenylated 3’-untranslated region (3’UTR) (400-700 nucleotides long) which is capable to form secondary stem-loop structure (1). The most 3’-terminal part of the 3’UTR is conserved and may be critical for the initiation of minus-strand RNA synthesis, whereas more proximal parts located downstream termination codon UAA are highly variable with functions being not completely revealed (1-3). Deletions introduced into the 3’UTR of different flaviviruses altered the infectivity of the mutants and reduced the efficiency of RNA replication (2, 3). Presumably, the 3’UTR variable region could function as an enhancer of viral RNA replication. The computer-assisted theoretical analysis showed that most of the deletions disrupted conserved secondary structure of the flavivirus 3’UTR (2). The tick-borne encephalitis virus (TBEV), being prevalent member of *Flaviviridae* family in Russia and Europe, causes severe neuroinfections with significant morbidity and mortality (1). Currently, the species is subdivided into 3 genetic subtypes: Far-Eastern, Siberian and European. The aim of the present work was to compare the 3’UTR variable fragments of different TBEV strains isolated from ticks during periods with different tick-borne encephalitis annual rate among patients.

## MATERIALS AND METHODS

The TBEV strains have been isolated from unfed imago of *Ixodes persulcatus* Schulze in Novosibirsk region, South-Western Siberia, Russia during period 1980–2006 as previously described (4). Hemagglutination titers of the TBEV strains were determined as earlier published (5). Incubation periods for suckling mice were analyzed as in (4). Molecular hybridization with radioactive oligonucleotide probes was performed as in (6). RNA isolation and RT with random hexamer followed by PCR were performed as in (5) with the following primers flanking variable fragment of the TBEV 3’UTR: 3’UTRd (5’-GAGTCCAAACTGGAGAGCTC-3’) and 3’UTRr (5’-ATGTTCCCGTCGCCACTCTC-3’). Nucleotide sequences of the RT-PCR products purified by gel electrophoresis were determined using Big Dye™ dideoxynucleotide Terminator kit and the ABI PRISM™ 310 Genetic Analyzer (Perkin Elmer Applied Biosystems, USA) at the DNA Sequencing Centre of Siberian Branch of the Russian Academy of Sciences, Novosibirsk, Russia (GenBank accession numbers DQ473396-DQ473404). The nucleotide sequences were aligned using MegAlign v 6.0 program of “DNAstar” software (DNASTAR Inc. 2004) based on CLUSTALW algorithm.

## RESULTS

The TBEV strains have been isolated from unfed imago of *Ixodes persulcatus* Schulze in Novosibirsk region, South-Western Siberia, Russia for 1980–2006 during periods with various sickness rates among people in the same endemic region.

Analysis of genetic variability of 79 TBEV strains was initially performed using molecular hybridization of the viral RNA with radioactive oligodeoxyribonucleotide probes complementary to different areas of the TBEV RNA. One of the most variable parts among different viral strains studied appeared to be 3’UTR (data not shown). Subsequent detailed analysis of genetic variability of 10 TBEV strains isolated during periods with maximal and minimal morbidity was carried out by RT-PCR with subsequent sequencing. All of the isolated strains belonged to Siberian genetic subtype (7) based on analysis of both E gene fragment sequences as previously described (4) and 3’UTR homology (data not shown).

**Figure 1:**
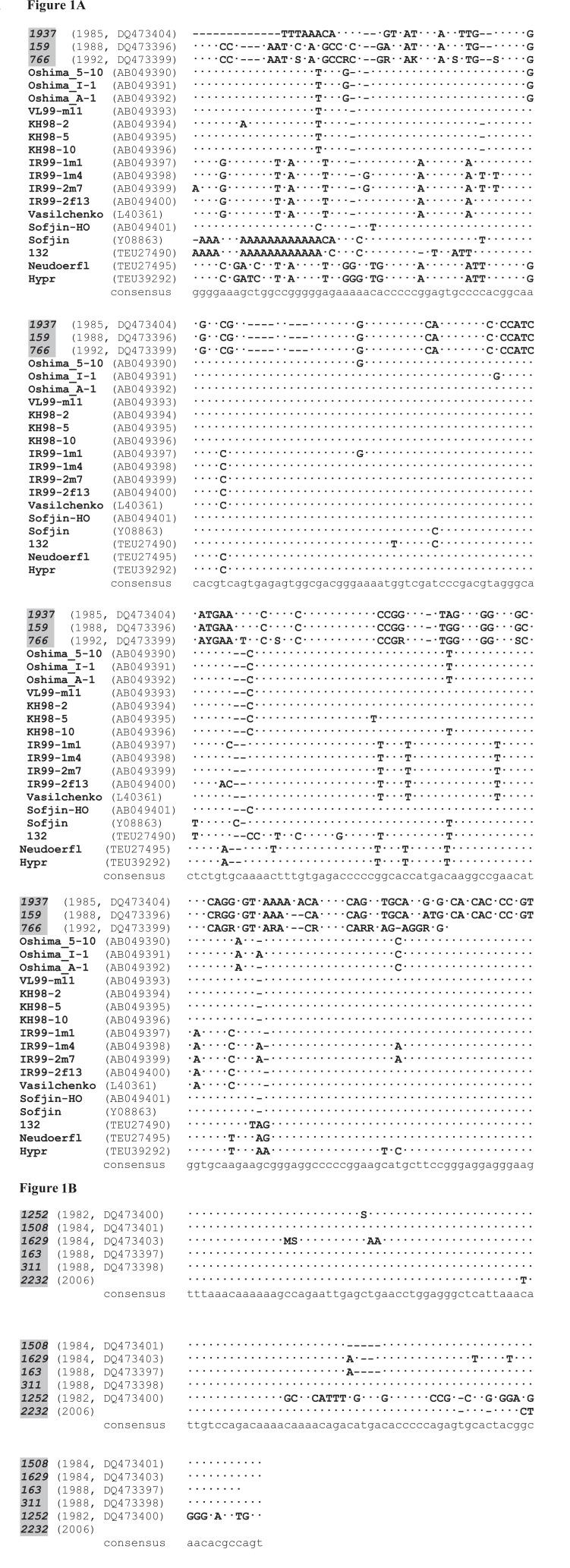
(A), Alignment of the TBEV 3’UTR nucleotide sequences homologous to previously described TBEV 3’UTR sequences. Names of strains sequenced in the present study are bold italic in gray shadows, previously known sequences are bold. Year of the strain isolation and GenBank accession number are given in parenthesis when available. Nucleotides identical to consensus (low case bottom line) are replaced by dots; (B), Alignment of the 3’UTR nucleotide sequences of TBEV strains with no significant homology to previously described TBEV 3’UTR sequences. Names of strains sequenced in the present study are bold italic in gray shadows. Year of the strain isolation and GenBank accession number are given in parenthesis when available. Nucleotides identical to consensus (low case bottom line) are replaced by dots.

Lengths of the RT-PCR products corresponding to 3’UTR variable fragment varied in a range 100-400 bp. Some strains had 3’UTR of certain length whereas majority of the studied strains especially among recently isolated strains after one or a few passages produced RT-PCR products of different lengths (data not shown). Alignment of 3’UTR nucleotide sequences revealed nucleotide substitutions, oligo (A)-tracts of 2-6 nucleotides long and microdeletions of 1-5 nucleotides (Fig. 1). Moreover, the nucleotide sequence alignment revealed two groups with significant homology within each group (Fig 1A, 1B) and essential differences between them. One group appeared to be homologous to the previously described TBEV 3’UTR sequences (Fig. 1A), while another did not reveal significant similarity with the known structures (Fig. 1B). It is interesting to note that one of the strain studied – TBEV 1252 (DQ473400) had unique 44 bp sequence (Fig. 1B).

**Figure 2:**
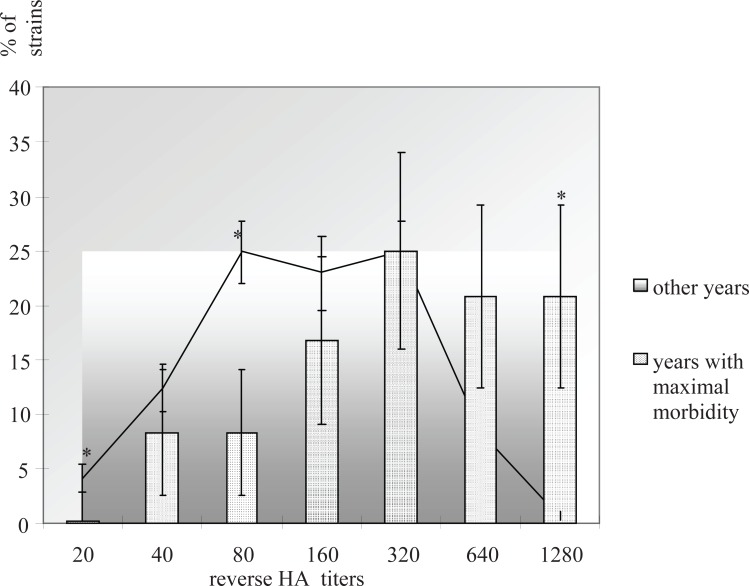
HA titers of the TBEV strains during periods with different tick-borne encephalitis rate (*P<0.05).

Hemagglutination (HA) titers for 241 strains were varied in a range from 1:20 to 1:1280 (Fig. 2). Correlation between HA titers and lengths of 3’UTR fragment was not found. However, cyclic variations of the TBEV HA titers coincided very well with periodic changes of the disease rate in humans. Neuroinvasiveness indexes for 40 TBEV strains varied in a range 0.3-4.3 (titers 5.8-8.5 lgLD_50_ after intracerebral infection and 2.7-7.0 lgLD_50_ after subcutaneous administration). It is interesting to note that in years with peak sickness rate among people the neuroinvasiveness indexes of the TBEV strains studied were higher than in other years.

## DISCUSSION

One of the striking epidemiological features of TBEV has been periodic variation in the occurrence and severity of TBEV infections in different endemic regions from Far Eastern Russia to Europe. Peak values last 1-2 years and trough values last for 6-7 years separated by intervals of gradual transition over 1-5 years. Four such cycles have been observed in the Novosibirsk region, South-Western Siberia, Russia spanning a period of 28 years beginning in 1980. The tick-borne encephalitis morbidity in Novosibirsk region periodically changed with maximal sickness rate in 1981 (47.7 incidences per 100,000 population), 1992 (60.7 incidences per 100,000), 1999 (32.9 incidences per 100,000) and 2007 (data not available). The TBEV infection rate of the main arthropod vector - *Ixodes persulcatus* ticks was previously shown to vary from 0.3 to 3.1 % according to bioassays (5) and up to 46 % based on RT-PCR (8). The TBEV strains isolated from ixodid ticks during period between 1980 and 2006 years in the same endemic region of Siberia, Russia were analyzed after 1-15 passages in susceptible laboratory suckling mice. Changes in TBE incidence in patients paralleled variations in the TBEV strains isolated from unfed ticks as determined by viral hemagglutination titers and by neuroinvasive indexes in newborn mice. The 3’UTR turned out to be one of the most variable parts of the genome. Sequencing revealed numerous 3’UTR rearrangements including microdeletions of 1-5 nucleotides located near oligo (A)-tracts 2-6 nucleotides long. The first group of the TBEV strains (Fig. 1A) included the high-virulent strains isolated in 1982 and 1992 with high morbidity among people in Novosibirsk region whereas all the TBEV strains from the second group (Fig. 1B) have been isolated during periods with relatively low sickness rate in the endemic region (1984, 1988 and 2006). Diversity observed for the 3’UTR sequences appeared to be not essential for the viability of the TBEV strains. Hemagglutination titers varied from 1:20 to 1:1280 for the TBEV strains and did not correlate with length of 3’UTR fragment. Longer incubation period for more than 5 days in suckling mice observed for the TBEV strains with shorter 3’UTR fragment sequences (less than 200 nucleotides) (Fig. 1B) in comparison with strains with longer (more than 300 bp) sequences (Fig. 1A) (4 days incubation period) might be probably caused by slower rate of RNA replication. However, reduced neuroinvasiveness can not also be excluded as the reason for different incubation periods. Thus, structural rearrangements in the TBEV 3’UTR presumably did not significantly affect translation but probably could compromise RNA synthesis.
